# Generalized myokymia, or neuromyotonia, or both in dogs with or without spinocerebellar ataxia

**DOI:** 10.1111/jvim.16892

**Published:** 2023-10-31

**Authors:** An Vanhaesebrouck, Mario Van Poucke, Kimberley Stee, Nicolas Granger, Edward Ives, Iris Van Soens, Ine Cornelis, Kenny Bossens, Luc Peelman, Luc Van Ham, Sofie F. M. Bhatti

**Affiliations:** ^1^ Queen's Veterinary School Hospital Cambridge University Cambridge UK; ^2^ Department of Veterinary and Biosciences, Faculty of Veterinary Sciences Ghent University Merelbeke Belgium; ^3^ Small Animal Department, Faculty of Veterinary Sciences Ghent University Merelbeke Belgium; ^4^ Bristol Vet Specialists CVS Referrals Bristol UK; ^5^ Anderson Moores Veterinary Specialists Winchester UK; ^6^ Companion Animal Clinic, Department of Clinical Sciences, Faculty of Veterinary Medicine University of Liège Liege Belgium; ^7^ Orion Veterinary Clinic Herentals Belgium

**Keywords:** canine, EAST syndrome, hereditary ataxia, peripheral nerve hyperexcitability

## Abstract

**Background:**

*KCNJ10* and *CAPN1* variants cause “spinocerebellar” ataxia in dogs, but their association with generalized myokymia and neuromyotonia remains unclear.

**Objective:**

To investigate the association between *KCNJ10* and *CAPN1* and myokymia or neuromyotonia, with or without concurrent spinocerebellar ataxia.

**Animals:**

Thirty‐three client‐owned dogs with spinocerebellar ataxia, myokymia neuromytonia, or a combination of these signs.

**Methods:**

Genetic analysis of a cohort of dogs clinically diagnosed with spinocerebellar ataxia, myokymia or neuromyotonia. *KCNJ10* c.627C>G and *CAPN1* c.344G>A variants and the coding sequence of *KCNA1*, *KCNA2*, *KCNA6*, *KCNJ10* and *HINT1* were sequenced using DNA extracted from blood samples.

**Results:**

Twenty‐four Jack Russell terriers, 1 Jack Russell terrier cross, 1 Dachshund and 1 mixed breed with spinocerebellar ataxia were biallelic (homozygous) for the *KCNJ10* c.627C>G variant. Twenty‐one of those dogs had myokymia, neuromyotonia, or both. One Parson Russell terrier with spinocerebellar ataxia alone was biallelic for the *CAPN1* c.344G>A variant. Neither variant was found in 1 Jack Russell terrier with ataxia alone, nor in 3 Jack Russell terriers and 1 Yorkshire terrier with myokymia and neuromyotonia alone. No other causal variants were found in the coding sequence of the investigated candidate genes in these latter 5 dogs.

**Conclusion:**

The *KCNJ10* c.627C>G variant, or rarely the *CAPN1* c.344G>A variant, was confirmed to be the causal variant of spinocerebellar ataxia. We also report the presence of the *KCNJ10* c.627C>G variant in the Dachshund breed. In dogs with myokymia and neuromyotonia alone the reported gene variants were not found. Other genetic or immune‐mediated causes should be investigated to explain the clinical signs of these cases.

**Abbreviations**
BAEP: brain auditory evoked potentials
CDS: coding sequence
EMG: electromyogram

## 1 INTRODUCTION

Generalized myokymia and neuromyotonia are most commonly observed in humans as an immune‐mediated disorder, in which there is an immune response against proteins (CASPR2 or LG1) tightly complexed to voltage‐gated potassium channels (Kv1.1, 1.2 and 1.6 encoded by *KCNA1*, *2* and *6*) of the peripheral nerve.^1^, ^2^ Generalized myokymia and neuromyotonia are described in hereditary disorders, related to variants in the genes encoding certain potassium channels of the peripheral nerves (*KCNA1* and *KCNQ2*).^3^, ^4^ Myokymia or neuromyotonia might also rarely accompany neuropathies, such as *HINT1* neuropathy.^5^

In young Jack Russell and Parson Russell terriers, there is a syndrome characterized by a typical bouncing, dancing gait.^6^ Histopathological features included degenerative axonal changes and demyelination within the spinal cord, brainstem, and peripheral nerves. This syndrome was termed “hereditary ataxia,” but is often now referred to as “spinocerebellar ataxia.”

Since 2004, several case series report that Jack Russell terriers with spinocerebellar ataxia often have signs of myokymia or neuromyotonia.^7^, ^8^, ^9^ *KCNJ10* c.627C>G variant is linked to Jack Russell and Parson Russell terriers with spinocerebellar ataxia.^10^ Seventy‐one percent of these dogs also have myokymia, 36% neuromyotonic episodes, and 21% seizures. The same *KCNJ10* variant is also found in Fox and Patterdale terriers with identical signs.^11^, ^12^ Spinocerebellar ataxia in a small number of Jack or Parson Russell terriers might be explained by a different *KCNJ10* variant (g.22141027insC).^13^ A third *KCNJ10* variant (c.986T>C) causing ataxia is reported in dog breeds unrelated to Terriers, such as Malinois dogs and Bouvier des Ardennes,^14^, ^15^, ^16^ further strengthening the association between variants in this gene and spinocerebellar ataxia. Two of the 3 affected Malinois dogs showed myokymia, 2 dogs developed neuromyotonic episodes (unpublished observation, KS) and 1 dog had a seizure. No myokymia was observed in any of the affected Bouvier dogs, although 1 dog had an episode resembling neuromyotonia.

Variants in *KCNJ10* are widely reported to explain spinocerebellar ataxia in affected dogs, a variant in another gene, *CAPN1* (c.344G>A), is reported in Parson Russell terriers presenting with spinocerebellar ataxia without myokymia, neuromyotonia or epilepsy.^17^ This variant could not be found in a later study examining a much larger group of ataxic Parson Russell terriers in Germany.^13^

Rare cases of generalized myokymia, neuromyotonia or both, without concurrent spinocerebellar ataxia are reported in Jack Russell terriers, Yorkshire terriers and 1 Border Collie.^8^, ^9^, ^18^, ^19^ The etiology of myokymia/neuromyotonia in these dogs is unclear and their possible association with *KCNJ10* variants has not been investigated.

In humans, *KCNJ10* variants are linked to “Epilepsy Ataxia Sensorineural Deafness Tubulopathy” (EAST) syndrome^20^ and *CAPN1* variants to spastic ataxia/paraplegia.^21^ Neither myokymia nor neuromyotonia is reported in association with *KCNJ10* or *CAPN1* variants in humans or in experimental animal models.^22^ This study aims to clarify the potential association between variants in *KCNJ10* and *CAPN1* genes and myokymia and neuromyotonia in a cohort of 33 dogs with spinocerebellar ataxia, myokymia/neuromyotonia, or both.

## 2 METHODS

### 2.1 Cases

The study was approved by the Ethical committees at Ghent University (EC2012/037) and Cambridge University (CR46). Dogs presenting with signs of spinocerebellar ataxia with or without myokymia or neuromyotonia were recruited from 4 referral centers across Belgium (1 dog was from the Netherlands), France and the United Kingdom between 2007 and 2014.

### 2.2 Phenotypic characterization

Spinocerebellar ataxia was defined as a dancing bouncing gait, involving all limbs, with a concurrent head “bobbing.” Myokymia was defined as vermicular movements of the skin or mucosa overlying the muscle, present in more than 2 muscles.^23^ Neuromyotonia was defined as a long‐lasting generalized muscle stiffness, causing the dog to lay down in lateral recumbency (because of limb rigidity) for more than 10 minutes, without any loss of consciousness and often associated with hyperthermia.^23^ These episodes can be preceded by facial rubbing. Generalized tonic‐clonic epileptic seizures were defined as an episodic event of short duration (less than 5 minutes), with loss of consciousness and commonly associated with hypersalivation, urination or defecation. A standard questionnaire was used during history taking for cases presented to Ghent University to gather information from the owner regarding the occurrence of myokymia, neuromyotonia, epileptic seizures and facial rubbing. In addition, videos of the events, recorded by the owner or veterinarian, were used to determine the phenotype. The phenotype of the dogs was confirmed by a boarded neurologist. In most dogs the diagnosis of myokymia or neuromyotonia was made clinically by observation of myokymia or neuromyotonia during the consult or on video recordings. Electromyogram (EMG) to confirm myokymia/neuromyotonia was performed in 15 dogs. Neuromyotonic discharges were defined as motor unit action potential discharges with an intraburst frequency of more than 150 Hz.^9^, ^24^ In nearly all dogs that underwent EMG, Brain Auditory Evoked Potentials (BAEP) and Electroneurogram were also performed. Serum biochemistry was performed, including measurements of potassium, calcium and magnesium and creatine kinase. Imbalances in serum electrolyte concentrations were excluded in all dogs.

### 2.3 Genetic analysis

Genomic DNA was isolated from EDTA‐blood samples. Sanger sequencing was used to genotype the *KCNJ10* XM_038448705.1:c.627C>G (p.(Ile209Met)) variant and the *CAPN1* XM_038425033.1:c.344G>A (p.(Cys115Tyr)) variant.^10^, ^17^ Dogs in which the phenotype of peripheral nerve hyperexcitability could not be explained by either of the 2 tested variants, were screened for causal variants in the coding sequence of candidate genes *KCNA1*, *KCNA2*, *KCNA6*, *KCNJ10* and *HINT1* via Sanger sequencing.^15^, ^25^ Details of the genetic analysis is given in Supplementary Info S1.

## 3 RESULTS

Thirty‐three dogs presenting with signs of spinocerebellar ataxia, myokymia or neuromyotonia were included (28 Jack Russell terriers, 1 Jack Russell cross, 1 Parson Russell terrier, 1 Yorkshire terrier, 1 Dachshund, and 1 mixed breed). Clinical and electrodiagnostic characteristics of 10 of these dogs are described, however their clinical description had not been linked to the reported genetic variants.^8^, ^9^, ^26^ Twenty‐one dogs presented with both spinocerebellar ataxia as well as myokymia, neuromyotonia or both, 8 dogs with ataxia alone, and 4 dogs with only myokymia and neuromyotonia (without ataxia; Table 1).

**TABLE 1:** Information on the breed, clinical phenotype (spinocerebellar‐like ataxia and myokymia/neuromyotonia) and genotype (*KCNJ10* and *CAPN1*) of the 33 investigated dogs.

Breed	SCA	M/NM	KCNJ10	CAPN1	Number
JRT	+	+	**G/G**	G/G	19^a^
Dachshund	+	+	**G/G**	G/G	1^b^
Mixed breed	+	+	**G/G**	G/G	1
JRT	+	−	**G/G**	G/G	5
JRT cross	+	−	**G/G**	G/G	1
PRT	+	−	C/C	**A/A**	1
JRT	+	−	C/C	G/G	1
JRT	−	+	C/C	G/G	3^c^
YT	−	+	C/C	G/G	1

*Note*: Variant alleles are in bold.

Abbreviations: *CAPN1*, XM_038425033.1:c.344G>A (p.(Cys115Tyr)) *CAPN1* genotype; JRT, Jack Russell terrier; *KCNJ10*, XM_038448705.1:c.627C>G (p.(Ile209Met)) *KCNJ10* genotype; M/NM, myokymia and/or neuromyotonia; PRT, Parson Russell terrier; SCA, spinocerebellar ataxia; YT, Yorkshire terrier.

^a^ Eight dogs were clinically described in reference 9.

^b^ This dog was described in reference 26.

^c^ One dog was described in reference 9.

All 21 dogs with spinocerebellar ataxia with myokymia or neuromyotonia were biallelic (homozygous) for the *KCNJ10* c.627C>G variant (19 Jack Russell terriers, 1 Dachshund and 1 mixed breed; Table 1). In addition, 6 of the 7 Jack Russell terriers (including 1 Jack Russell terrier cross) had spinocerebellar ataxia without myokymia or neuromyotonia were biallelic for this *KCNJ10* variant. The only Parson Russell terrier, also suffering from spinocerebellar ataxia, did not carry this *KCNJ10* variant, but was biallelic for the *CAPN1* c.344G>A variant. One Jack Russell terrier with only ataxia, and all 4 dogs with myokymia and neuromyotonia without ataxia (3 Jack Russell terriers and 1 Yorkshire terrier) did not carry either of these 2 genetic variants. Coding sequence analysis of *KCNA1*, *KCNA2*, *KCNA6*, *KCNJ10* and *HINT1* in these 5 dogs did not reveal any other causal variant. The clinical signs by gene variant are summarized in Table 2.

**TABLE 2:** Information on the breed, age, clinical signs and genotype of the 33 investigated dogs.

Breed (N)	Age (m)	Age of onset (m)	SCA	M	NM	Epilepsy	Facial rubbing	Hyperthermia	Exercise/stress‐related	CK average (range)	Variant
JRT (24)	11	6	100%	74%	63%	19%	44%	33%	56%	880	*KCNJ10*
JRT cross (1)	(3–24)	(2–11)	(27/27)	(20/27)	(17/27)	(5/27)	(12/27)	(9/27)	(15/27)	(42‐2000)	
Dachshund (1)											
Mixed breed (1)											
PRT (1)	40	30	100%	0%	0%	0%	0%	0%	0%	101	*CAPN1*
			(1/1)	(0/1)	(0/1)	(0/1)	(0/1)	(0/1)	(0/1)		
JRT (1)	30	30	100%	0%	0%	0%	0%	0%	0%	599	None
			(1/1)	(0/1)	(0/1)	(0/1)	(0/1)	(0/1)	(0/1)		
JRT (3)	59	23	0%	100%	100%	0%	0%	25%	50%	493	None
YT (1)	(12‐144)	(6‐60)	(0/4)	(4/4)	(4/4)	(0/4)	(0/4)	(1/4)	(2/4)	(299‐687)	

Abbreviations: CK, creatine kinase (reference, <321 IU/L); JRT, Jack Russell terrier; M, clinical myokymia; NM, clinical neuromyotonia; PRT, Parson Russell terrier; SCA, spinocerebellar ataxia; YT, Yorkshire terrier.

Clinical apparent myokymia with or without neuromyotonia is pathognomonic for peripheral nerve hyperexcitability, and can be confirmed by EMG. Neuromyotonic discharges were recorded in limb, trunk and facial muscles of 77% of dogs biallelic for the *KCNJ10* variant (n = 10/13; described in detail in references 9, 26). These discharges were typically recorded when myokymia was also visible during recordings, and were not recorded in a dog with spinocerebellar ataxia alone. Although myokymia was not clinically, neuromyotonic discharges were recorded in the tongue muscle of the Parson Russell terrier biallelic for the *CAPN1* variant. In 1 dog presenting for myokymia/neuromyotonia alone, EMG was silent, with no myokymic muscle contractions visible during recordings. Electroneurogram was normal in all dogs tested. Delay and/or absence of central BAEP waves (described in detail in^9^, ^26^) were found in all dogs biallelic for the *KCNJ10* variant, but not in the Parson Russell terrier biallelic for the *CAPN1* variant,^9^, ^26^ nor in a dog with myokymia/neuromyotonia alone.

Most dogs presenting with clinically overt myokymia and neuromyotonia had concurrent spinocerebellar ataxia (84%, 21/25), with all of those dogs being biallelic for the reported *KCNJ10* variant (Table 2). In the study cohort, isolated myokymia and neuromyotonia without spinocerebellar ataxia appeared in only 4 dogs. The clinical manifestation of myokymia (Video S1) and neuromyotonia (Video S2) in those 4 dogs was identical to myokymia and neuromyotonia that occurs in association with ataxia in dogs biallelic for the *KCNJ10* variant.

Wormlike movements under the skin (myokymia) were described by the owners of all 4 dogs with isolated myokymia and neuromyotonia. In 2 of those dogs, myokymia was witnessed during the consultation and in another 2 they were confirmed by video recordings. This muscle rippling was usually observed in scapular, gluteal and biceps muscles and in 1 dog also around the eyes. Myokymia occurred either episodic (3 dogs) or continuously (1 dog). In 2 Jack Russell terriers the occurrence of myokymia was related to stress, excitement or exercise. In the Yorkshire terrier, myokymia started approximately 1 hour before a neuromyotonic episode. Neuromyotonic episodes were described in all 4 dogs, during which they were in lateral recumbency, showed generalized muscle rigidity; they remained conscious and had no autonomic signs. The stiffness was most pronounced in the pelvic limbs in 2 dogs, and in 1 dog (the Yorkshire terrier) the thoracic limbs were more affected. A high temperature was recorded in 1 dog, and heavy panting was reported in 2 other dogs during these episodes. The episodes of stiffness could last from 20 minutes to up to 16 hours, and their frequency ranged from once every couple of days to 5 episodes a year. In 2 dogs, there was a relationship between neuromyotonic episodes and exercise, excitement or stress. Facial rubbing or seizures were not described in any of the dogs. In 1 of the dogs, the signs improved over time. There was no knowledge of other littermates affected. Dogs with isolated, myokymia and neuromyotonia were significantly older at presentation (average 4.9 years versus 0.9 years), compared to dogs biallelic for the *KCNJ10* variant, and the onset of signs appeared later in life (average of 23 months versus 6 months; Table 2).

## 4 DISCUSSION

This study supports the strong association between “spinocerebellar ataxia” and genetic variants in *KCNJ10* and *CAPN1*—the latter variant was later called into question^13^—in a cohort of dogs from Belgium, the Netherlands, France and the United Kingdom.^10^, ^17^ We now also report the *KCNJ10* c.627C>G variant causing spinocerebellar ataxia in the Dachshund breed. Although clinical myokymia and neuromyotonia was common in dogs with spinocerebellar ataxia and biallelic for the *KCNJ10* variant (21 out of 27 dogs in this study, 78%), a proportion of such dogs (6 out of 27 dogs, 22%) did not have signs of myokymia and/or neuromyotonia. In addition, dogs presenting only with myokymia or neuromyotonia (without ataxia) did not carry the reported *KCNJ10* or *CAPN1* variants (4 dogs). This opens the debate as to whether these variants are causally related to the myokymia and neuromyotonia observed in the affected dogs. In addition, this study confirms that the recording of neuromyotonic discharges in limb muscles^9^, ^10^ and BAEP abnormalities in dogs with spinocerebellar ataxia^9^, ^26^, ^27^ are associated with the *KCNJ10* variant.

The *KCNJ10* c.627C>G variant was confirmed to be the causal variant in nearly all affected dogs with clinical signs of spinocerebellar ataxia, except for 1 dog in which the *CAPN1* variant was detected and another dog for which the diagnosis remains unknown. The *KCNJ10* variant, already described in Jack Russell terriers, Parson Russell terriers and Fox terriers,^10^, ^11^ was not only found in 24 Jack Russell terriers, but also in 1 Jack Russell terrier cross, a mixed breed, and a Dachshund. Based on the mixed breed physical appearances, Jack Russell terrier ancestry seemed likely, which could explain the origin of the variant. In the Dachshund with spinocerebellar ataxia, this is the first report of the presence of this variant in this breed. Although the exact origin of the Dachshund breed remains unclear, they were most likely crossed with terriers in the past,^28^ which could also explain the presence of this variant in the breed. The *CAPN1* c.344G>A variant, described in Parson Russell terriers,^17^ was confirmed to be the causal variant in 1 affected Parson Russell terrier and was not found in any of the Jack Russell terriers, in accordance to literature.^13^

That these known causal variants were not found in 4 dogs (3 Jack Russell terriers and 1 Yorkshire terrier) that presented with classical myokymia and neuromyotonia signs, but lacking ataxia—a manifestation described by several authors^8^, ^9^, ^18^, ^19^—suggests that isolated, generalized myokymia and neuromyotonia might be caused by another genetic variant or that these dogs have an acquired syndrome, similar to humans.^2^ It is still not understood why not all dogs biallelic for *KCNJ10* variants develop myokymia or neuromyotonia and why myokymia or neuromyotonia is not reported in humans with EAST syndrome.

## CONFLICT OF INTEREST DECLARATION

Authors declare no conflict of interest.

## OFF‐LABEL ANTIMICROBIAL DECLARATION

Authors declare no off‐label use of antimicrobials.

## INSTITUTIONAL ANIMAL CARE AND USE COMMITTEE (IACUC) OR OTHER APPROVAL DECLARATION

Approved by the Ethical committees at Ghent University (EC2012/037) and Cambridge University (CR46).

## HUMAN ETHICS APPROVAL DECLARATION

Authors declare human ethics approval was not needed for this study.

## Supporting information


**Data S1.** Supplementary information.

**Video S1.** Jack Russell terrier showing myokymia in the right pelvic limb.

**Video S2.** Neuromyotonic episode in a Yorkshire terrier.
