# Supplementary material for: Generalized myokymia, or neuromyotonia, or both in dogs with or without spinocerebellar ataxia
Source: J Vet Intern Med. 2023 Oct 31;37(6):2310–4. doi: 10.1111/jvim.16892 (PMC10658559; doi:10.1111/jvim.16892)
Supplement: Supplementary file 1 — Data S1. Supplementary information. [file JVIM-37-2310-s002.pdf]

Supplementary Info 1. Detailed description of the molecular genetic analysis.

1. DNA isolation
2. PCR mixes and cycling conditions
3. Sanger sequencing
4. Genotyping assay for the KCNJ10 XM_038448705.1:c.627C>G (p.(Ile209Met)) variant
5. Genotyping assay for the CAPN1 XM_038425033.1:c.344G>A (p.(Cys115Tyr)) variant
6. Resequencing the coding sequence of candidate genes KCNA1, KCNA2, KCNA6, KCNJ10 and HINT1
7. References

1. DNA isolation

One hundred µl EDTA blood cells were washed 3 times with 500 µl TE (10 mM Tris-HCl, pH 8; 1 mM EDTA, pH 8) and digested for 50' at 56°C in 100 µl digestion buffer (10 mM Tris-HCl, pH 8; 50 mM KCl, 0.5% Tween 20) supplemented with 1 µl 20 µg/µl proteinase K. After inactivation of the proteinase K (10' at 95°C), cell debris was centrifuged (1' at 16,100 x g) and 2 µl of the DNA-containing supernatant was used to perform PCR.

2. PCR mixes and cycling conditions (Ta, annealing temperature)

```
PCR mix with TEMPase HS DNA Polymerase
 5.7 µl H2O
 1.0 µl 10x Key buffer
 1.0 µl Primer mix (5 µM each primer; IDT)
 0.2 µl dNTPs (10 mM each nucleotide)
 0.1 µl TEMPase HS DNA Polymerase (5 U/µl)
 2.0 µl Template
10.0 µl Total volume
```

```
PCR cycling conditions (S1000, Bio-Rad)
with TEMPase HS DNA Polymerase
14'30" - 95°C
00'30" - 95°C ]
00'30" - Ta°C ] x35
01'00" - 72°C ]
02'00" - 72°C
HOLD   - 15°C
```

3. Sanger sequencing

Two µl of the PCR product was validated via 2% agarose gel electrophoresis. The rest of the validated PCR product (a single amplicon of the correct length) was treated with 4 U Exonuclease I and 2 U Antarctic Phosphatase (Exo/AP; Biolabs) for 30' at 37°C. After enzyme inactivation (15' at 80°C), a Sanger sequencing reaction was performed with 2 µl of the Exo/AP-treated PCR product and an individual PCR primer as sequencing primer, using the BigDye Terminator v3.1 Cycle Sequencing Kit (Applied Biosystems). Sequencing reactions were run on an ABI 3730 XL sequencing machine at Eurofins Genomics and sequence analysis was performed with UGENE[1].

```
Sequencing mix (Applied Biosystems)
 3.0 µl H2O
 0.5 µl RR-mix
 2.0 µl 5x sequencing buffer
 1.0 µl GC-rich (Roche)
 1.5 µl Sequencing primer (2 µM)
 2.0 µl Exo/AP-treated or eluted  PCR product
10.0 µl Total volume
```

```
Sequencing cycling conditions (S1000, Bio-Rad)
02'00" - 95°C
00'20" - 95°C ]
00'15" - 60°C ] x30
04'00" - 65°C ]
HOLD   - 15°C
```

4. Genotyping assay for the KCNJ10 XM_038448705.1:c.627C>G (p.(Ile209Met)) variant[2]

Primers F1 (5'-CATCGGCTACGGCTTCCGCT-3') and R1 (5'-CAAGGGACTGGTCTCATCTACCACG-3') were used to amplify a variant containing fragment of 412 bp with TEMPase HotStart DNA Polymerase (Ta = 64°C), that was Sanger sequenced with the F1-primer.

5. Genotyping assay for the CAPN1 XM_038425033.1:c.344G>A (p.(Cys115Tyr)) variant[3]

Primers F2 (5'-GGGCTGGTTTGCCTAGATTCCTG-3') and R2 (5'-AGCTTTGGCCGTGTGGAACC-3') were used to amplify a variant containing fragment of 232 bp with TEMPase HotStart DNA Polymerase (Ta = 64°C), that was Sanger sequenced with the R2-primer.

6. Resequencing the coding sequence of candidate genes KCNA1, KCNA2, KCNA6, KCNJ10 and HINT1

KCNA1, KCNA2 and KCNA6 genes were resequenced as described in Van Poucke et al. (2012)[4]. KCNJ10 was resequenced as described in Van Poucke et al. (2017)[5]. Primers F3 (5'-ACCTCCCTTCTGCCGGGTCT-3') and R3 (5'-ACCCCTTCCCGTCCTTCCGT-3') were used to amplify a HINT exon 1 containing amplicon of 427 bp with TEMPase HotStart DNA Polymerase (Ta = 60°C), that was Sanger sequenced in both directions with the F3- and R3-primer. Primers F4 (5'-AGTGCTTCTCATGGGGCTAGTGT-3') and R4 (5'-ATCACTTTCAGGTCACAATTTAGTCTCCC-3') were used to amplify a HINT exon 2 containing amplicon of 489 bp with TEMPase HotStart DNA Polymerase (Ta = 64°C), that was Sanger sequenced in both directions with the F4- and R4-primer. Primers F5 (5'-TGTTACCACAGCTTAACCAAATCTAGCC-3') and R5 (5'-ACTTCCTCAATCTATCCATACACAGGCA-3') were used to amplify a HINT exon 3 containing amplicon of 610 bp with TEMPase HotStart DNA Polymerase (Ta = 64°C), that was Sanger sequenced in both directions with the F5- and R5-primer.

7. References

[1] Okonechnikov K, Golosova O, Fursov M; UGENE team. Unipro UGENE: a unified bioinformatics toolkit. Bioinformatics. 2012 Apr 15;28(8):1166-7. doi: 10.1093/bioinformatics/bts091.

[2] Gilliam D, O'Brien DP, Coates JR, Johnson GS, Johnson GC, Mhlanga-Mutangadura T, Hansen L, Taylor JF, Schnabel RD. A homozygous KCNJ10 mutation in Jack Russell Terriers and related breeds with spinocerebellar ataxia with myokymia, seizures, or both. J Vet Intern Med. 2014 May-Jun;28(3):871-7. doi: 10.1111/jvim.12355.

[3] Forman OP, De Risio L, Mellersh CS. Missense mutation in CAPN1 is associated with spinocerebellar ataxia in the Parson Russell Terrier dog breed. PLoS One. 2013 May 31;8(5):e64627. doi: 10.1371/journal.pone.0064627.

[4] Van Poucke M, Vanhaesebrouck AE, Peelman LJ, Van Ham L. Experimental validation of in silico predicted KCNA1, KCNA2, KCNA6 and KCNQ2 genes for association studies of peripheral nerve hyperexcitability syndrome in Jack Russell Terriers. Neuromuscul Disord. 2012 Jun;22(6):558-65. doi: 10.1016/j.nmd.2012.01.008.

[5] Van Poucke M, Stee K, Bhatti SF, Vanhaesebrouck A, Bosseler L, Peelman LJ, Van Ham L. The novel homozygous KCNJ10 c.986T>C (p.(Leu329Pro)) variant is pathogenic for the SeSAME/EAST homologue in Malinois dogs. Eur J Hum Genet. 2017 Feb;25(2):222-226. doi: 10.1038/ejhg.2016.157.
